# Z Factor: Drama as a tool to tackle mental health stigma: study design and protocol for community and public engagement in rural Zimbabwe

**DOI:** 10.12688/wellcomeopenres.16262.1

**Published:** 2021-02-08

**Authors:** Denford Gudyanga, Tamaryn Palmer, Nicola Wright, Eileen O'Regan, Charity Shonai, Nefasi Mlambo, Melody Maremera, Walter Mangezi

**Affiliations:** 1 Zimbabwe Early Intervention in Psychosis Project (Together We Thrive Trust), Harare, Zimbabwe; 2 Department of Psychiatry, Faculty of Medicine and Health Sciences, University of Zimbabwe, Harare, Zimbabwe; 3 School of Health Sciences, Faculty of Medicine, University of Nottingham, Nottingham, UK; 4 Nottinghamshire Healthcare NHS Foundation Trust, Nottingham, UK; 5 Ministry of Health and Child Care, Government of Zimbabwe, Harare, Zimbabwe; 6 Zimbabwe Open University, Harare, Zimbabwe

**Keywords:** Zimbabwe, Mental Health Stigma, Public Engagement

## Abstract

**Background: **Mental health is slowly gaining global significance as a key health issue, yet the stigma attached to psychosis is still a major problem. There has been little in-depth exploration of sustainable, cost-effective, and replicable community engagement strategies that address mental health myths and stigma, which are major barriers to early health-seeking behaviours. In low-income countries such as Zimbabwe, cultural and spiritual beliefs are at the centre of most mental health explanatory models, perpetuating an environment where mental health conversations are a cultural taboo. Mental health interventions should be accompanied by creative, evidence-based community engagement, ensuring that interventions are suitable for local settings and giving communities a voice in directing their health initiatives.

**Methods: **Z Factor aimed to engage young adults and their support networks across a variety of socioeconomic groups in a rural district of Zimbabwe through their participation in an inter-ward five-staged drama competition. The focus was on psychosis, with subcategories of initial presentation/detection, seeking help/pathway to care, and the road to recovery/treatment. Each drama group’s composition included a young adult and a typical support network seeking treatment from the service provider of choice. Dramas were to act as discussion starters, paving the way toward broader and deeper psychosis treatment discussions among rural communities and gaining insight into service user expectations from health research.

**Conclusions: **Outcomes of the pilot community engagement project will be instrumental in improving understanding community perceptions about psychosis treatment and recovery in rural Zimbabwe and increasing community awareness about psychosis, as well as paving the way for initiating service provider collaboration to promote early detection and encouraging early health-seeking behaviours. The above outcomes will also inform the design of models for more responsive community and public engagement initiatives in similar low resource settings in Zimbabwe and beyond.

## Introduction

Mental health is slowly gaining global significance as a key health issue ( Keyes, 2007; World Health Organization, 2004). This has not, however, resulted in a significant decrease in the stigma attached to psychosis, especially in low-income countries ( Kemp *et al.*, 2019). Neither has there been an in-depth exploration of sustainable, cost-effective and replicable community engagement strategies that address mental health myths and stigma as a major barrier to early health-seeking behaviours. In low income and resource-constrained countries such as Zimbabwe, cultural and spiritual beliefs are at the centre of most mental health explanatory models ( Refugee Review Tribunal AUSTRALIA, 2009). This perpetuates an environment in which public conversations about mental health are the equivalent of a cultural taboo ( Patel *et al.*, 1995; Wintersteen *et al.*, 1995). Mental health interventions should therefore be accompanied by creative ways of engaging communities with health research ( Walker, 2009). As public engagement is increasingly becoming an indispensable tool in promoting global health ( Adhikari *et al.*, 2019), it is helping to ensure that interventions are not only suited to the local setting but equally important, communities are given a voice in directing their health initiatives ( Nisker *et al.*, 2006). ****

Zimbabwean culture has always valued drama and its sub-components of music and dance as a crucial element in community socialization and fostering unity. Drawing from the experiences of health researchers from the African Mental Health Research Initiative (AMARI) and the University of Zimbabwe who have successfully engaged the public in creative ways, Z Factor, a drama competition modelled on the popular television music competition franchise (X Factor), combined music, dance and poetry as a socially acceptable medium of community socialization in Zimbabwe. This medium, which has never been adequately harnessed in mental health programmes, was adapted as a creative tool for public engagement with psychosis in rural Zimbabwe. The implementing organization, the Zimbabwe Early Intervention in Psychosis (ZimEIP) project, which has been working in rural Zimbabwe since 2016, sought to introduce a community arts and culture competition (Z Factor) as a creative way of opening up conversations, sparking public debate and tackling stigma attached to psychosis in rural Zimbabwe. Key outcomes for Z Factor include gaining a more in-depth appreciation of community perceptions towards psychosis treatment by health researchers, as well as increasing community awareness about psychosis.

As noted by the World Health Organization, there is a shortage of skilled mental health service providers in Zimbabwe, with 13 clinical social workers, six psychologists, 18 psychiatrists and 917 psychiatric nurses ( WHO, 2020). As such, Z Factor efforts were also directed towards mental service provider collaboration to promote early detection and treatment-seeking behaviours. This is especially important as current work by the ZimEIP project in rural Zimbabwe has identified a deeply embedded traditional belief system influencing how, when and where to seek mental health care. Refugee Review Tribunal Australia (2009); Patel *et al.* (1995) and Winston & Patel (1995) argue that these deeply embedded traditional beliefs attributing the causes of psychosis to evil spirits and witchcraft attacks is a recurrent theme across most of sub-Saharan Africa. Consequently, this has led to the stigmatization of psychosis, which in turn fuels poor health-seeking behaviours in African communities. It was, therefore, hoped that the psychosis themed drama competition would help to combat stigma by offering alternative explanations of psychosis as well as allowing the implementing organization (ZimEIP) and its mental health research collaborators to learn more about the community belief systems, with the hopes of identifying suggestions for further exploration in which the two mental health care pathways (traditional and biomedical) may collaborate towards a more robust and context responsive psychosis treatment model in Zimbabwe.

## Project objectives

The Z Factor project aimed to pilot the use of drama as a public engagement tool to open up community conversations about psychosis in Zimbabwe. This would be achieved through engaging young adults and their support networks across a variety of socioeconomic groups through their participation in a local drama competition to:

1. Reduce psychosis stigma and discrimination in rural Zimbabwe.

2. Describe the community’s perspective about psychosis treatment pathways and outcomes in rural Zimbabwe.

3. Develop collaborative care between the traditional/faith-based and medical models of psychosis treatment.

4. Develop a context-specific and community acceptable mental health public engagement model for low resource settings.

The drama competition, therefore, served a dual role as follows:

a) As a vehicle for opening up community conversations using the psychosis themed drama.

b) As a medium for psychosis treatment information exchange between service providers (traditional/ faith-based, biomedical practitioners) and service users (the local community, persons with psychosis and their support networks).

## Project setting

Goromonzi Rural District is approximately 40km from Zimbabwe’s capital city, Harare. According to Goromonzi Rural District Council (2020) and ZimStat (2012), the Goromonzi District of Zimbabwe is made up of 25 wards with a population of 224 987. Communal areas make up 53.17% of the district, 42.36% are commercial farming areas, and the remainder are semi-urban settlements ( ZimStat, 2012). The ZimEIP program has been working in two of the 25 wards of Goromonzi and these have a combined population of 22,647. ZimEIP has been working in two of the major district referral clinics, one of which houses the only psychiatric nurse in the district. The Z Factor project was therefore implemented in the two wards of Goromonzi where the implementing organization already had a presence since 2016 with an established mental health stakeholder network. As Goromonzi Rural District Council (2020) note that the population density of the district is approximately nine people per square kilometre, each ward can be made up of as many as 30 villages.

## Project design

**Figure 1.**
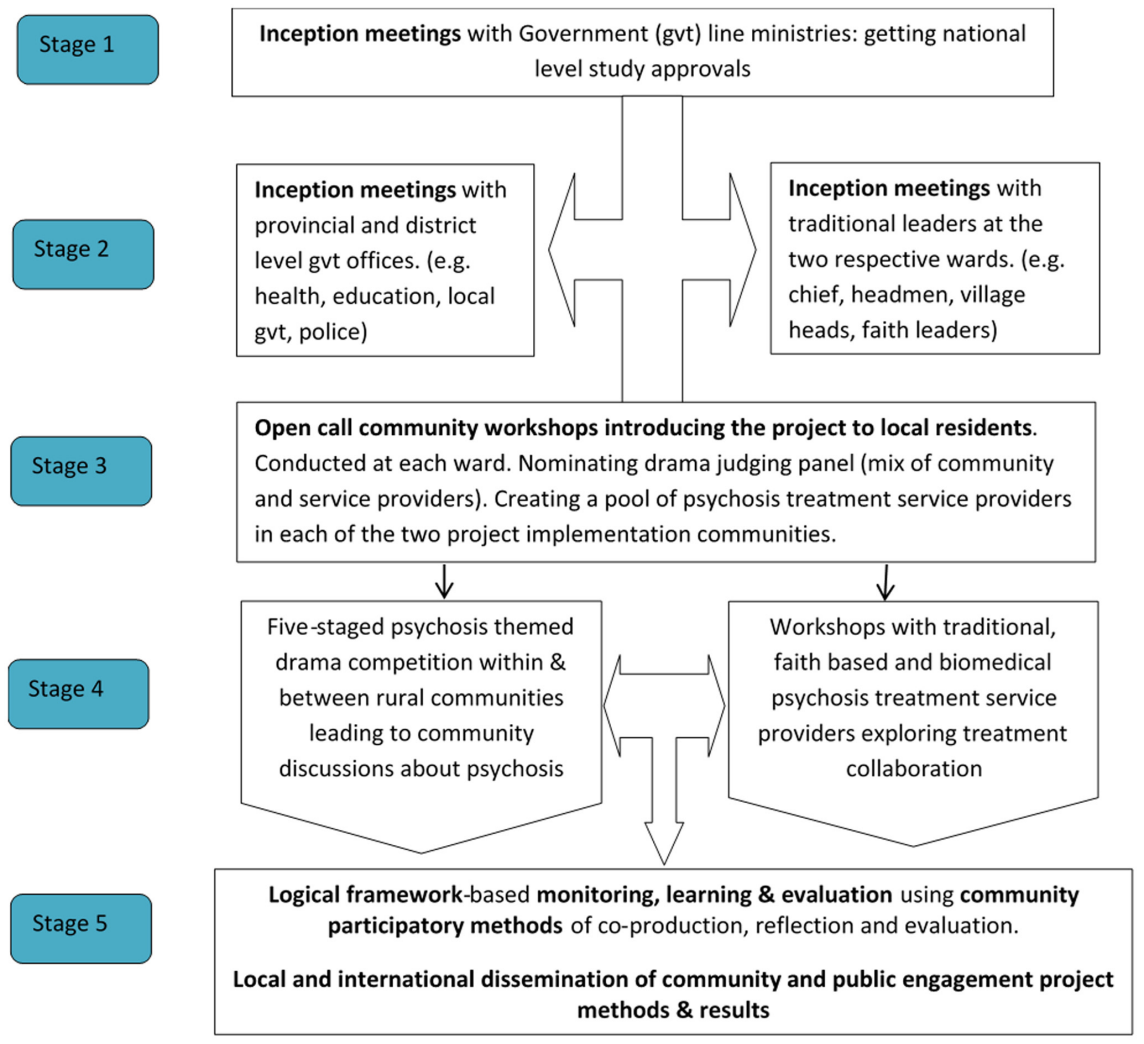
Proposed community and public engagement activity structure.

This community and public engagement project had a three-pronged approach. It sought to promote mental health conversations in rural communities by providing a fun and interactive platform for community discussions. These were designed to demystify psychosis by providing accurate treatment information from different mental health service providers, as well as explore a collaborative model for biomedical, traditional and faith-based service providers to work together towards reducing psychosis treatment delays. A five-staged psychosis themed drama competition (see Figure 1) was therefore implemented in the Goromonzi District of Zimbabwe where ZimEIP has been implementing its Early Intervention in Psychosis project since October 2016.

The competitions endeavoured to open up community conversations about psychosis in rural communities where mental health stigma is fuelled by cultural and religious explanatory models of mental health causes. Z Factor adopted a co-production model where both the research participants and mental health researchers work together in formulating and delivering the engagement project. A community participatory method was therefore adopted in planning, implementing and evaluation throughout the project’s life cycle as a way of encouraging participation and ownership. As visual methods have been noted to be a useful public engagement tool in HIV and other illnesses, the project also explored the use of drama as a creative and fun way of opening up psychosis conversations.

## Stakeholder consultation

Zimbabwe has a dual judicial and administrative system composed of the colonially inherited British system as well as the traditional system, which is more pronounced in rural districts ( Bennett, 1981). As Kurebwa (2018) observes, in Zimbabwe, both the central government departments and traditional chiefdom serve as community gatekeepers dictating the provision of social services in their areas. It was, therefore, crucial to get these gatekeepers’ buy-in as well as permission to implement the Z Factor project. Permission was therefore sought from the Ministry of Health and Child Care, Ministry of Local Government, Ministry of Home Affairs and the Ministry of Youth, the area chiefs, their headmen and village heads, the ward councillors and District Member of Parliament for Goromonzi among other relevant authorities in the area. These gatekeepers were visited individually as well as invited to a gatekeepers’ stakeholders meeting at the District Administrator’s office introducing the Z Factor project as well as getting their feedback on the proposed project implementation plan.

The village health workers in the rural sites chosen had prior training in early detection of Psychosis and appropriate patient referral system under the ZimEIP project. Efforts were made towards engaging and recruiting the two wards’ village health workers and village heads to be responsible for onsite planning and mobilizing participants. Feedback from these onsite planners and participant mobilisers were to be incorporated in further developing the project implementation plan. Feedback from the first stakeholders’ meeting with government ministerial heads was also intended to further refine the project implementation plan. This monitoring and evaluation strategy was intended to be a continuous process throughout the project life cycle where both project implementors and the participants would actively take part in co-planning and co-implementation. Selected and willing village health workers acting as community mobilizers were tasked with promoting the drama competition through village meetings and any other community forums with permission from local gatekeepers. Stakeholder consultation meetings with village heads and other traditional as well as church leaders were intended to assist village health workers to gain the necessary permission and support needed to mobilize communities. The drama competitions and discussion workshops were, therefore, to be advertised at village level meetings, church gatherings and other community gatherings. The competitions were also to be publicised in local schools, both primary and secondary as well as local health facilities. The disseminated information was to include contact details for community mobilisers available to provide more details on the proposed competitions, as well as the activities calendar and location.

## Public engagement drama competition structure

The project’s aim was engaging young adults and their support networks across a variety of socioeconomic groups in Goromonzi Rural District of Zimbabwe through their participation in a local drama competition (Z Factor). The competitions, therefore, focused on psychosis with subcategories of the initial presentation/detection, seeking help/pathway to care, and the road to recovery/treatment (see Table 1). Each drama group had to be composed of an adolescent/young adult and a representative of their support network i.e., family members, friends, neighbours and teachers. The drama and drama group composition was therefore intended to act as a reflection of society. Further, each drama group had a 10-minute slot with a limit on the number of groups per event (see Table 1) as more time was to be allocated to follow up discussions soon after the drama exhibitions.

**Table 1.** Proposed Z Factor drama competition structure and topics.

** Drama auditions** No specific theme/topic: pick any topic about psychosis (kurwara nepfungwa/hurwere hwepfungwa) Maximum of 15 groups per ward to make it into stage 1 (30 groups in total) Each group should be allocated 10 minutes			
**Stage**	**Chinyika ward**	**Drama topic**	**Rusike ward**
**1st Drama** **Competition**	Maximum of 10 groups per ward to make it into stage 2	*How is someone with psychosis identified* * in your community (i.e. what is the initial * *presentation and how is it detected)?*	Maximum of 10 groups per ward to make it into stage 2
**2nd Drama ** **competition**	Maximum of six groups per ward to make it into the 1st combined stage	*What is it like to live with a young person* *with psychosis in a family and how is that* *family treated in your community?*	Maximum of six groups per ward to make it into the 1st combined stage
**Combined inter-ward drama competition** *To be held at a centralized community centre selected by the community and public engagement participants*			
**1 ^st^ combined** ** Drama** ** Competition**	**Theme:** *At what stage do people, individuals or families seek help about psychosis in your community?*		
	Maximum of 10 groups to make it into the second stage		
**2 ^nd^ combined** ** Drama** ** Competition**	**Theme:** *How is psychosis treated in your community and who treats it? Show us where families and * *individuals seek help about psychosis issues in your community*		
	Maximum of seven groups to make it into the last stage		
**Final competition and presentation of prizes to the winners**			
**Final competition**	**Theme:** *Show us how traditional medicine, churches and biomedical clinics can work together in treating* * psychosis in your community*		
	Three groups to win the competition for 1st 2nd and 3rd prizes, the losing four groups to get consolation prizes		

The work of the implementation organization (ZimEIP) in the Goromonzi communities has revealed that family elders’ i.e. parents, uncles, aunties and grandparents are key stakeholders in deciding the pathway to care for the ill family member, as well as being better positioned to influence early health-seeking behaviours. Therefore, to be in sync with the cultural norms in Zimbabwean communities that highly venerate community elders, with assistance from the ZimEIP Team, local clinic personnel and drama experts, community elders were appointed as part of the judging panel as a way of ensuring their active involvement in the public engagement as well as encouraging community ownership of the activities.

The competitions were open to auditions whereby an adolescent or young adult and their potential support networks were invited to take part and compete within and between wards and villages. Community members and stakeholders in attendance voted for who should proceed to the next level based on the accuracy to which their dramatizations mirrored community psychosis perceptions and social norms. Break-out groups were to run parallel to the competitions as a platform for in-depth discussion between community members, mental health service stakeholders and ZimEIP personnel as an aid to the drama voting system. These competitions were conceived as an incentive for the general public to attend, as drama can be dually entertaining and informative. Discussion workshops were to follow through as a platform for further community engagement exploring themes brought out from the drama. The drama competition was designed to act as a common factor that brings together the whole community, from churches to African traditionalists, school students and families to enjoy themselves and open up the conversation about psychosis. Interested drama groups were not given any scripts to use but a theme/topic to dramatize what is happening in their communities and this would be judged by a trained panellist of community elders chosen during the first community meeting. These dramatizations that reflect what is happening in the community would act as conversation starters, paving the way for a more interactive discussion where service providers would also share accurate cultural and biopsychosocial information of mental health treatment and recovery within the Zimbabwean context. 

## Intended project beneficiaries

The project aimed to benefit the Chinyika and Rusike communities; it did not have any exclusion criteria as it sought to engage the community as a whole. Beneficiaries can, however, be classified into the following groups: individuals/community members, mental health service providers and organizations/policymakers within these communities. Further, as communities begin opening up about mental health conversations and the biases they may have towards persons with a mental health condition, it is hoped that such discussions may perhaps pave the way towards addressing and/or eradicating mental health stigma and discrimination, which are argued by Dharitri *et al.* (2015) to be one of the causes of poor health-seeking behaviours in communities.

### Benefits to participants

*Community members:* aimed to provide a platform where communities have a chance to reflect upon their own beliefs and perceptions about psychosis and the available treatment pathways, as well as the benefits of information exchange between service providers from different fields (biomedical, faith healing & traditional medicine). This intended networking was designed to empower communities with knowledge of where and how to get help if needed, as well as breaking down barriers between different psychosis treatment pathways, thereby helping to reduce mental health stigma and encourage early health-seeking behaviours, argued by Corrigan *et al.* (2014) to be severely hampered by mental health stigma.

*Service providers:* aimed to provide a platform where service providers can interact with their service users, gaining important insights into the challenges and expectations of psychosis treatment users as well as exploring a collaborative model for the biomedical, faith and traditional services to work together towards reducing treatment delay as well as mental health stigma and discrimination.

*Psychosis treatment users & support networks:* it is hoped that by demystifying psychosis conversations in rural communities through encouraging open discussions about mental health, the community can be made to reflect upon its own stigmatizing beliefs and work towards improving the treatment outcomes for persons with psychosis through the creation of a tolerant and accepting community ( World Health Organization, 2013). To that end, mental health service users and their support systems in the two wards of rural Goromonzi would meet with mental health service providers and policymakers during a focused group discussion as part of Z Factor project activities. Through this discussion platform, it was hoped that service users would have an opportunity to influence the provision of mental health services in their communities.

*Policymakers:* As the project involved the participation of policymakers at various levels both in the government and traditional chiefdom, it was hoped that they will be able to interact with mental health service users and their support networks with the hopes that the engagement can spark the need to prioritize mental health issues in policy programming. This is especially important as various studies and reports about Zimbabwean mental health services ( Chikara & Manley, 1991; Hendler *et al.*, 2016; Kidia, 2018; and Mangezi & Chibanda, 2010) argue that the country still has a long way to go in providing adequate mental health services due to lack of adequate financial and human resources as well as poor and negative belief systems about psychosis. It is therefore important that policymakers be actively involved in mental health research as such involvement provides them with first-hand knowledge and lived experiences of persons and or families affected by mental illness.

*Competition prizes:* As an incentive to taking part in the drama competitions, prize money and food hampers were to be given to both the winning as well as the losing drama groups at the inter ward level. The winning group would receive USD400, with the second group getting USD300 and lastly the third group getting USD200 in cash prizes. The losing four groups would each receive food hampers worth USD75 per group. Additionally, each group would receive refreshments per each activity as well as branded bags and or T-shirts which would also assist in community mobilization. 

## Monitoring and evaluation

The project’s monitoring and evaluation plan was informed by AMARI’s community and public engagement resources, as well as the University of Zimbabwe’s Research Support Centre. The logical framework approach was chosen to guide the planning, implementation and evaluation of the project as it is a widely used model for coming up with “indicators against which the project progress and achievements can be assessed” ( Barreto Dillon, 2020). Logical framework matrixes are argued by USAID (2011) to foster adherence to overall project aim yet are flexible enough to anticipate potential risks and threats, as well as incorporating learning to improve effectiveness from one project implementation stage to the next. Anticipated threats included poor attendance due to mental health stigma as well as geographical accessibility of project activity sites. Incentives such as refreshments and, where appropriate, transport fare reimbursement had to be budgeted for each engagement activity from dramas to discussion workshops. Transport reimbursements were to be given on a means-tested basis as well as mobilizing available transportation from community well-wishers. The logical framework approach was therefore adopted as a useful tool to track various project indicators to measure progress and allow for change management from one project phase to the next.

Due to the mental health stigma in the community of implementation, the project anticipated an overall turnout of 400 participants. This was based on data from mental health programmes carried out by other organizations and attendance statistics which were available at the District Administrator’s as well as local clinics’ databases.

## Data analysis

### Data sources (a)

Activity reports, audio/video recordings of activities, attendance registers and pictures and project implementation monitoring and evaluation field notes. Questionnaires, judges score sheets and any other documented materials collected during the project implementation phase. This excludes data from the end of project evaluation which is presented under a separate heading.

### Data sources (b)

The following activities were conducted during the project implementation phase as an important component of the community engagement project, parallel to drama competitions and or as standalone activities on separate days to the competitions.

*Focused group discussions:* with purposively sampled mental health service users (patients and their caregivers), key stakeholders made up of community elders who are also gatekeepers as well as mental health service providers (made up of faith healers and Christian pastors, traditional healers and herbalists, the Muslim herbalist community as well as clinic staff, village health workers and health researchers). These discussions aimed to provide a platform for information exchange between service users and providers, as well as exploring potential mental health research priority areas and of collaboration between traditional and biomedical services.

Data were to be collected through the above described 12 focus group discussions, six per ward. The discussions were to take place primarily in Shona as it was the preferred local language with English as an optional language. Convenience sampling was to be used to select participants per each ward, focusing on those available and willing to give their insights and experience regarding mental health stigma in the two communities and as well as potential ways of combating stigma and promoting social inclusion of persons with or recovering from mental health conditions. Potential participants would be contacted through the District Administrator’s, Traditional Medical Practitioner’s and local clinics’ databases of health providers and service users and well as local religious organizations. Thereafter a snowballing technic would be used to invite more participants to the community engagement activities.

*Key informant interviews:* with mental health service users, drama group leaders, village heads attending project activities, clinic nurses and the area/district Headmen. Stakeholders from various organizations (e.g. nurses, faith healers, traditional healers and local government officers). Semi-structured interviews would be conducted with people who participated in the Z Factor public engagement project. Convenience sampling, focusing on those available and willing to be interviewed, will be used to select participants.

Facilitation guides were to be used during focus group discussions and semi-structured interviews sessions. Additionally, all focus group discussions and interviews would be audio-recorded and transcribed verbatim during data analysis. Where the discussion switched between the local language, Shona and English, the appropriate parts of the transcript would be translated and checked for contextual quality and accuracy through back translation where appropriate. Summary notes of the discussion were also be taken by the activity facilitator. Line by line coding will be used to develop themes based on content analysis by a group of qualitative analysis experts. The initial codes will be generated by a group of qualitative researchers from the University of Zimbabwe’s Research Support Centre.

*Scaled questionnaire:* a simple Likert scale type questionnaire was to be administered to 15 drama groups pre-and post-event as well as during the end of project evaluation exercises. The participant would rate various segments of the Z Factor project on a 5 or 10 scale point. All drama groups were to be invited to complete the brief survey questionnaire assessing their mental health attitudes and their opinion towards using drama as a stigma reduction tool. Drama groups were purposively chosen as the group easiest to follow up throughout the project life cycle.

### Data analysis and presentation

All quantitative data will be analysed with appropriate statistical packages recommended by the project research collaborators who have done similar projects and these will be presented graphically. Further, the project evaluation questionnaire responses will be analysed using SPSS and outputted as simple descriptive statistics. This will be performed by the Harare based team with support from the University of Zimbabwe Research Support Centre. No personal identifying information will be captured.

Qualitative data, which makes for the bulk of the data collected, will be analysed based on themes using content analysis’ line by line coding and presented thematically. Members of the University of Zimbabwe Research Support Centre and other project collaborators will assist in transcribing, translating, coding and or analysis of the data as well as preparing manuscripts for dissemination.

### Data storage

All project data is stored on external CDs/DVDs, laptops hard drives and hardcopy document files in a locked steel cabinet. However, depending on appropriateness, all efforts are being made to ensure that project data is always backed up to Google Drive to minimize the risk of losing data.

### End of project evaluation

The end of project evaluation was carried out by the Z Factor project team with support from the University of Zimbabwe’s Research Support Centre. Participants who took part in the evaluation were provided with refreshments and reimbursed for transport costs. Local clinics were used as the focal point for meeting end of project evaluation participants; however, efforts were made to accommodate participants who could not easily access the clinic for various reasons.

The project evaluation methods are described below.

Project monitoring: The evaluation sought to describe the process of delivering the Z factor public engagement project. This was to include basic demographic information from participants (e.g., age, gender) if available, number of and attendance at activities, as well as the cost of running the Z factor public engagement project.

Qualitative data: Focus groups and semi-structured interviews were to be used to evaluate the perceived benefit of the Z factor public engagement project using focused group discussions and interviews as follows:

- Focus group discussions were to be conducted with a total of 4 groups. Each group were to have an estimated 10 participants of the Z factor public engagement project from Chinyika and Rusike wards in Goromonzi.
- Two group discussions were to be conducted per ward with community mobilisers, drama judging panellists, activity facilitators and participants. It was hoped that these focus groups would give insights on the challenges of organizing and facilitating the public engagement activities and the potential benefits of the project, and changes observed in the communities throughout both the project and evaluation exercise 6 months post-project activities.
- Semi-structured interviews were to be conducted with 20 people who participated in the Z factor public engagement project as mental health service users, their caregivers or service providers. 10 will be from each ward focusing on those available and willing to be interviewed.

Quantitative data: Both the focus group and semi-structured interview participants were to be asked to complete a brief self-administered end of the project survey questionnaire ( Gudyanga, 2021). The Project evaluation Questionnaire will be used to quantify participants’ understanding of the project goal, assess its success and effectiveness as well as making recommendations. These will be graphically presented.

Evaluation participants: Participants in the evaluation will be purposively sampled focusing on satisfying the following preconditions.

Inclusion criteria:

•   Individuals who participated in and or facilitated the Z Factor project activities were drama actors and community mobilisers.

•   Convenience sampling was to be used to select 50 participants per ward who were both available and willing to give their insights and experience participating in the Z Factor activities. As the exercise aimed to highlight participants’ views on the perceived challenges and benefits of taking part in the project.

•   Individuals who can provide informed consent to have their views recorded and or shared as outcomes of the evaluation.

Exclusion criteria:

•   Individuals who did not participate or facilitate the Z Factor activities, such as people who were not drama actors, community mobilisers or community discussion participants.

•   Individuals who are unable and or unwilling to provide informed consent to have their views recorded and or shared as outcomes of the evaluation

## Ethical considerations

### Project implantation approval

Permission and clearance have been sought from The Ministry of Health and Child Care’s Provincial and District level offices as well as the Joint Research Ethics Committee for the University of Zimbabwe and Parirenyatwa Group of Hospitals (JREC 169/19). As the project is of a pilot nature, allowances for the evolution of the design and methods throughout the project life span will be made. As such, the final evaluation tools will be submitted to JREC for approval before the dissemination of project outputs and outcomes.

### Informed consent

Both group and individual informed consent forms were given to drama actors and other participants that agreed to be recorded. However, declining to sign the consent forms would not affect participation in the community and public engagement activities as the project was open to all community members and had no exclusion criteria. More so, selected drama skits that convey useful themes about psychosis treatment in rural Zimbabwe were to be re-enacted following re-administering of the consent forms at end of the project evaluation phase. At various stages, participants were asked to re-consent as the anticipated content of the activities pre- and post-activity would likely change. Hence participants were allowed to re-consent to have their audio and visual recordings publicly disseminated.

People who participated in the Z Factor public engagement project as drama actors, community mobilisers and activity facilitators, as well as community discussion participants, were to be invited to take part using the project evaluation consent form (see *Extended data*) ( Gudyanga, 2021). The consent form was to be available in both English and Shona translations. Participants were to be given time to read through, ask questions and consider their involvement in the evaluation exercise. It was to be made clear that involvement in the focus group discussion or interviews is voluntary and should they choose not to take part, their relationship with the University of Zimbabwe or the Z factor project will not be affected. Further participants who would be willing to contribute their views off record would have their opinions captured under field notes.

### Managing mental health stigma

A decision was made not to censor drama groups on the nature of the dramatization content following a given psychosis treatment theme/topic. This would ensure that the drama themes portray what is truly happening in the rural communities, therefore acting as a springboard for sparking in-depth community discussions. It is hoped that by uncensored drama scripts, the community will be allowed to reflect upon their own beliefs and perceptions regarding mental illness.

As the project anticipated that there was always a potential risk of increasing stigma and or having people reliving unpleasant past experiences through taking part in these community events depicting lived experiences, persons living with psychosis or any other mental health conditions were to be part of the focus group discussions and wider community discussions but were not be required or coerced to participate as drama actors unless if they volunteered and/or received support from their families and friends. Further, the project ensured that both lay and qualified counsellors and a mental health nurse were available to offer support to those who needed it. This was hoped to be able to sufficiently guard against causing undue trauma or exacerbating the stigmatization of persons with psychosis. However, persons with psychosis and/or any other mental health conditions were to have an opportunity for exchanging knowledge, beliefs and experiences with various mental health service providers both at the wider community discussion level as well as dedicated workshops and focused group discussions.

### Participant safety

As the bulk of the project activities were to be convened at public places, clearance to invite and convene public gatherings were to be sought from the Zimbabwe Republic Police and the Local Government through the office of the District Administrator. Police Officers were to be assigned to monitor and promote public safety at the community and public engagement events. Community and public engagement activities would, therefore, take place at approved venues and times per Government of Zimbabwe laws.

### Cost of participation

 As highlighted by Goromonzi Rural District Council (2020), Goromonzi communities are spaced out with approximately nine people per square kilometre. The Z Factor community and public engagement activities were, therefore, to be conducted at ward level where each ward can have as many as 30 villages, thereby guarantying greater attendance numbers. However, drama actors, judging panellists and other stakeholders were either to be provided transport to community and public engagement venues or reimbursed for their travel costs. This, however, was to be done on a case by case basis as activities were to be convened at centralized and already established ward community centres. Furthermore, the project team were to make efforts towards sourcing out transportation donations from community stakeholders to reduce project costs.

## Discussion

Like in many sub-Saharan countries, rural communities in Zimbabwe have shown a general lack of accurate information regarding psychosis and its treatment, compounded by the traditional and religious explanatory models ( Refugee Review Tribunal AUSTRALIA, 2009). These models largely attribute the causes of psychosis to witchcraft and evil spirits, which in turn fuels stigmatization in communities ( Winston & Patel, 1995). As a result, not only are there delays in seeking treatment by communities, there is often dual consultation, diagnosis and treatment as people often seek treatment from traditional/faith as well as biomedical services, and this has often fuelled hostilities between these seemingly incompatible service providers. Mike Bestor *et al.* (2016) therefore argues that creative public engagement that promotes a “two- way communication that is interactive and continuous intending to share decision- making power and responsibility for those decisions” should inform strategies and programmes that seek to develop communities. Such “two-way communication” in public engagement is therefore needed to understand societal beliefs and offer mutually acceptable and culturally sensitive alternative evidence-based explanations for psychosis that promote early health-seeking behaviours. This also provides the potential to share accurate bio-psychosocial treatment information and encourage communities to reduce the delay in their health-seeking behaviours, with hopes to pave way for the two pathways to care (traditional/faith-based and the biomedical model) to collaborate in diagnosis and treatment management of mental illness in rural Zimbabwe.

Collins *et al.* (2012) argue that the link between mental health and poor health-seeking behaviours can be broken through promoting early intervention services. This is achieved through combating mental health stigma noted to be at the core of poor health-seeking among mental health service users ( Collins *et al.*, 2012). The knowledge gaps about psychosis stigmatization and the need to encourage timely health-seeking have necessitated the need for creative methods of public engagement ( Weetra *et al.*, 2019). We, therefore, aimed to provide a platform that attracts public attention ( Coyle *et al.*, 2017), opening up conversations and sparking public debate about psychosis in ways in which communities feel comfortable expressing their true beliefs and experiences regarding psychosis. It was hoped that this public engagement would see both the implementing organization Zimbabwe Early Intervention in Psychosis (ZimEIP) and the Goromonzi community learning from each other to pave the way for collaboration towards promoting community mental health in communities with deeply entrenched traditional and religious beliefs. The engagement was to ride on the commitment pledged by the chieftain and other traditional and faith leaders in Goromonzi towards the promotion of community mental health.

## Dissemination

Project outcomes and outputs will be shared with project participant groups in the form of lay summaries and, where requested, drama group participants will be provided with DVDs of their dramas and exhibitions. Manuscripts of the community and public engagement study design, methods and/or evaluation outcomes, as well as any other significant project outcomes/outputs, will be submitted to appropriate open access journals for wider dissemination.

## Study status

Initial project activities began in November 2017 and data collection ended in July 2018. Currently (20/11/2020), project data analysis is underway.

## Data availability

### Underlying data

No underlying data are associated with this article.

### Extended data

Harvard Dataverse: Replication Data for: Z Factor: Drama as a tool to tackle mental health stigma study in Zimbabwe”, https://doi.org/10.7910/DVN/GBRGL4 ( Gudyanga, 2021).

This project contains the following extended data:

-   Extended data 1 - Community Attitudes -Z Factor project- English + Shona.pdf

-   Extended data 2 - Z Factor Drama Judges scoring sheet.pdf

-   Extended data 3a - Z factor project pre-post event Community Knowledge Questionnaire.pdf

-   Extended data 3 b - Z factor project community attitudes questionnaire.pdf

-   Extended data 3c - Generic Z factor project planning questionnaire.pdf

-   Extended data 4 – Z factor project Focus group guide - agenda items.pdf

-   Extended data 5 – Z factor project Informed Consent Form - general.pdf

-   Extended data 6 - Z Factor Consent Form - Project Evaluation.pdf

-   Extended data 7a - Z Factor Project Evaluation tools - Survey.pdf

-   Extended data 7b - Z Factor Project Evaluation tools - Interview guide.pdf

-   Extended data 7c - Z Factor Project Evaluation tools - Focus group discussion.pdf

-   Extended data 8 - Z Factor project Logical framework.pdf

-   Data are available under the terms of the Creative Commons Zero “No rights reserved” data waiver (CC0 1.0 Public domain dedication).

## Disclaimer

The views expressed in this publication are those of the author(s) and not necessarily those of the Wellcome Trust.

## References

[ref-1] Adhikari B Vincent R Wong G: A realist review of community engagement with health research [version 2; peer review: 4 approved, 1 approved with reservations]. *Wellcome Open Res.* 2019;4:87. 10.12688/wellcomeopenres.15298.2 31289754 PMC6611131

[ref-16] Barreto Dillon L: Logical Framework Approach SSWM - Find tools for sustainable sanitation and water management!2020. Reference Source

[ref-2] Bennett TW: Conflict of Laws: The Application of Customary Law and the Common Law in Zimbabwe. *The International and Comparative Law Quarterly.* 1981;30(1):59–103. Reference Source

[ref-18] Bestor M Davison C Hart P: Creative Public Engagement Methods. [Government & Nonprofit].2016. Reference Source

[ref-3] Chikara F Manley MR: Psychiatry in Zimbabwe. *Hosp Community Psychiatry.* 1991;42(9):943–947. 10.1176/ps.42.9.943 1743667

[ref-4] Collins RL Wong EC Cerully JL: Interventions to Reduce Mental Health Stigma and Discrimination: A Literature Review to Guide Evaluation of California’s Mental Health Prevention and Early Intervention Initiative.2012;47. 10.7249/TR1317 PMC5052078 28083275

[ref-5] Corrigan PW Druss BG Perlick DA: The Impact of Mental Illness Stigma on Seeking and Participating in Mental Health Care. *Psychol Sci Public Interest.* 2014;15(2):37–70. 10.1177/1529100614531398 26171956

[ref-6] Coyle K Lowry S Saunders J: See Change: The National Mental Health Stigma Reduction Partnership in Ireland. In: W. Gaebel, W. Rössler, & N. Sartorius (Eds.), *The Stigma of Mental Illness—End of the Story?*Springer International Publishing.2017;357–377. 10.1007/978-3-319-27839-1_19

[ref-7] Dharitri R Rao SN Kalyanasundaram S: Stigma of mental illness: An interventional study to reduce its impact in the community. *Indian J Psychiatry.* 2015;57(2):165–173. 10.4103/0019-5545.158175 26124523 PMC4462786

[ref-9] Goromonzi Rural District Council: Goromonzi Rural District Council.2020. Reference Source

[ref-10] Gudyanga D: Replication Data for: Z Factor: Drama as a tool to tackle mental health stigma study in Zimbabwe. Harvard Dataverse, V1,2021. 10.7910/DVN/GBRGL4 PMC7931254 33693064

[ref-11] Hendler R Kidia K Machando D: "We Are Not Really Marketing Mental Health": Mental Health Advocacy in Zimbabwe. *PLoS One.* 2016;11(9):e0161860. 10.1371/journal.pone.0161860 27607240 PMC5015838

[ref-12] Kemp CG Jarrett BA Kwon CS: Implementation science and stigma reduction interventions in low- and middle-income countries: a systematic review. *BMC Med.* 2019;17(1):6. 10.1186/s12916-018-1237-x 30764820 PMC6376798

[ref-13] Keyes CLM: Promoting and protecting mental health as flourishing: a complementary strategy for improving national mental health. *Am Psychol.* 2007;62(2):95–108. 10.1037/0003-066X.62.2.95 17324035

[ref-14] Kidia KK: The future of health in Zimbabwe. *Global Health Action.* 2018;11(1):1496888. 10.1080/16549716.2018.1496888 30058477 PMC6070968

[ref-15] Kurebwa J: The Institution of Traditional Leadership and Local Governance in Zimbabwe. * Int J Civ Engagem Soc Change.* 2018;5(1):1–22. 10.4018/IJCESC.2018010101

[ref-17] Mangezi W Chibanda D: Mental health in Zimbabwe. *Int Psychiatry.* 2010;7(4):93–94. 10.1192/S1749367600006032 31508055 PMC6734988

[ref-19] Nisker J Martin DK Bluhm R: Theatre as a public engagement tool for health-policy development. *Health Policy.* 2006;78(2–3):258–271. 10.1016/j.healthpol.2005.10.009 16337306

[ref-20] Patel V Mutambirwa J Nhiwatiwa S: Stressed, Depressed, or Bewitched? A Perspective on Mental Health, Culture, and Religion (Stressé, déprimé, ou ensorcelé? Une vue sur la santé mentale, la culture et la religion / Tensão, depressão ou fascinação? Uma perspectiva em saúde mental, cultural e religiosa / Estresado, deprimido o hechizado? Una perspectiva en salud mental, cultura y religión). *Dev Pract.*JSTOR.1995;5(3):216–224. Reference Source

[ref-21] Refugee Review Tribunal AUSTRALIA: Zimbabwe - Mental illness - Social attitudes - Treatment programs - Support groups - Legal provisions. *RRT RESEARCH RESPONSE.* 2009;23. Reference Source

[ref-22] USAID: USAID Project Design Guidance.2011. Reference Source

[ref-23] Walker E: Learning through action: Engagement and behavioural change through the use of drama. *Development and Learning in Organizations.* 2009;23(6):18–21. 10.1108/14777280910994877

[ref-24] Weetra D Glover K Miller R: Community engagement in the Aboriginal Families Study: Strategies to promote participation. *Women Birth.* 2019;32(1):72–79. 10.1016/j.wombi.2018.04.002 29699794

[ref-25] WHO: Zimbabwe WHO Special Initiative for Mental Health Situational Assessment.2020. Reference Source

[ref-26] Winston CM Patel V: Use of traditional and orthodox health services in urban Zimbabwe. *Int J Epidemiol.* 1995;24(5):1006–1012. 10.1093/ije/24.5.1006 8557433

[ref-27] Wintersteen RT Mupedziswa R Wintersteen LB: Zimbabwean Families of the Mentally 111: Experiences and Support Needs.1995;19.

[ref-28] World Health Organization: Promoting Mental Health.World Health Organization,2004. Reference Source

[ref-29] World Health Organization: WHO Collaboration - one answer to strengthening mental health services in Zimbabwe.WHO. n.d., Retrieved January 21, 2020. Reference Source

[ref-30] World Health Organization: WHO Investing in mental health.WHO.2013. Reference Source

[ref-31] ZimStat: ZIMBABWE POPULATION CENSUS 2012.2012;187. Reference Source

