# Supplementary material for: Z Factor: Drama as a tool to tackle mental health stigma: study design and protocol for community and public engagement in rural Zimbabwe
Source: Wellcome Open Res. 2021 Feb 8;6:26. [Version 1] doi: 10.12688/wellcomeopenres.16262.1 (PMC7931254; doi:10.12688/wellcomeopenres.16262.1)
Supplement: Peer review history

# Peer review: Reviewer response for version 1

**DOI:** 10.21956/wellcomeopenres.17866.r42570

**Published:** 2021-03-03

**Authors:** Mary Chambers

**Affiliations:** 1 Oxford University Clinical Research Unit, University of Oxford, Ho Chi Minh City, Vietnam

In this article the authors outline the rationale and methods for a pilot community engagement project to aid understanding of community perceptions about psychosis and health seeking behaviour in rural Zimbabwe. A co-production approach was used to design a multiple-stage drama competition that aimed to create a platform for discussions in the community, raise suggestions for improving early health-seeking behaviour and inform researchers and service-providers of barriers to seeking care and community priorities.

 The article is very clearly structured, methods are well described and extended data usefully includes templates of documents used in the project and for evaluation.

 This article makes a valuable contribution to the field of community engagement, and community-based approaches to addressing mental health, particularly in lower and lower middle income settings.

 In the introduction the authors state that "this medium has *never* been adequately harnessed..." I acknowledge there are few reports of such projects, which makes the publication of the Z-Factor project even more timely, however  I refer them to The Theatre for Positive Mental Health (https://epale.ec.europa.eu/en/blog/theatre-positive-mental-health-project-supported-erasmus) as one example and suggest they change the word *never* to *rarely*.

 They record the value of the competition prizes but I suggest they give an indication of a typical wage in those regions so the readers can gauge the relative value in those communities. 

 I look forward to reading the project evaluation report.

Is the study design appropriate for the research question?

Yes

Is the rationale for, and objectives of, the study clearly described?

Yes

Are sufficient details of the methods provided to allow replication by others?

Yes

Are the datasets clearly presented in a useable and accessible format?

Yes

Reviewer Expertise:

Public and community engagement and participatory visual methods.

I confirm that I have read this submission and believe that I have an appropriate level of expertise to confirm that it is of an acceptable scientific standard.

# Peer review: Reviewer response for version 1

**DOI:** 10.21956/wellcomeopenres.17866.r42571

**Published:** 2021-02-11

**Authors:** Pradeep Narayanan

**Affiliations:** 1 Research Division, Praxis Institute for Participatory Practices, New Delhi, Delhi, India

I congratulate the authors of *“Z Factor: Drama as a tool to tackle mental health stigma: study design and protocol for community and public engagement in rural Zimbabwe”* for this delightful commentary on the project, while elucidating with abundant detail on objectives, rationale and activities of the project. The project itself is innovating in amalgamating the two phenomena: social acceptance of drama as a medium and social rejection of psychosis through stigmatisation.

**Study Rationale: **As I understand, the Project has made certain significant assumptions based on review of the literature. From amongst global focused literature, the project assumed or deciphered: *one,* the global significance of Mental health issue is growing but not resulting in a significant decrease in the stigma attached to psychosis, especially in low-income countries; *two,* community engagement models on mental health issues are lacking; and *three* **, **public engagement models are becoming indispensable for health interventions. From amongst literature on Zimbabwe, the project assumed or discovered: *four* **,** Zimbabwean *culture* values drama and it is *socially acceptable* medium of community socialization, and therefore a medium to be used for social messaging on stigmatisation; *five* **,** presence of deeply embedded traditional belief system influencing mental health seeking behavior of members; *and* traditional beliefs attributing the cause of psychosis to evil spirits and witchcraft attack, leading to stigmatization of mental health.

 In all this, there is also a reference to a shortage of skilled mental health service providers in Zimbabwe, and therefore need to promote early detection and treatment-seeking behaviours.

 Keeping the above five assumptions in mind, the rationale of the project is to use a socially accepted medium to facilitate public engagement on a culturally stigmatized phenomenon of psychosis, with the objective: **one,** to reduce psychosis stigma; **two,** to develop collaborative care between the traditional faith based and medical models; and **three,** thereby, arrive at a context-specific and community acceptable mental health public engagement model for low resource settings.

 The rationale is no doubt convincing, and the best part of the project is that the project would embark into stimulating the five assumptions in its journey, especially those related to the ground situation in Zimbabwe. At this stage, the key to the success of the project would be, I believe, in its ability to understand, whether stigmatization of mental health is exclusively linked to it being deeply entrenched in traditional healing system or is it linked to absence of empathy for persons living with mental illness, which causes communities to go for any healing that is available; or both. Further, is collaborative care conditioned on certain terms, imposed by medical models; or are even those terms open for engagement? The challenge for the project is its ability to be *sincere* in its participatory methods, for the method it has chosen would allow diverse information- politically correct and incorrect, scientific and non-scientific; socially acceptable and non-acceptable- enter into the domain of information exchange; and the worst that could happen is when the project, with its own baggage of what it represents, would filter and sanitise those information and reject a lot even before they mutate. I am sure, the project would reflect on what and who it represents; and how monolith it is, It is important for the project to know whether it has transformed over the period or not. A discussion on this reflection would enrich the article.

**Study design:** The study design is an embodiment of participatory action research. An intensive inception meeting with all important stakeholders, followed by open call community workshops to facilitate drama auditions and creating systems and platforms for community involvement and engagement. Then the study embarks into psychosis themes drama competitions ensuring community members participate not only as audience, but also as actors, story-writers, judges and others. Ensuing spaces for peer-learning and participatory reflection by different stakeholders, there are also simultaneous workshops with service providers exploring collaborations. The crucial aspect of participatory research is to ensure that the creator of data uses the data, and the project getting enriched and informed by used data; and additionally having datasets even on use of used data. The stage 5 of the design is very crucial for that becomes the litmus test for the Project to infer on how participatory the project has been; merely being participatory in ‘project activities’ does not make the Project participatory. The crucial miss, generally, in the public engagement model is its inability to engage the public in measuring the strength of public engagement, and thereby causing instrumentalising of public and of engagement, rather than making engagement intrinsic to the model. The fear often is that projects, generally, learn about this dimension only at the stage of post-mortem.

**Project details facilitating replication: **The article has an amazing amount of details. I read through the entire article with interest. It is very clear and provides very important details. To be frank, after reading this article, I am very tempted to use this approach in one of the intervention areas that I am involved with. Keeping that selfish interest in mind, I would feel the article would be enriched if it has the following information. Firstly, I believe there are multiple power relationships that are operating at the societal level: medical versus traditional healers; traditional healers versus faith-based healers; government officials versus traditional chief; bureaucrats versus people; among faith leaders; and between faith and people. The mental health belief system that is present now is an outcome of these power relationships. When the project says that it got the ‘buy-in’, what does it mean in the real sense? It would be good to ‘problematise’ this buy-in and present that as threat or as opportunity or as both.

 Secondly, in the same way, the use of ‘drama’, which is being a socially acceptable medium, for messaging on socially non-accepted phenomena like psychosis: how it actually interacts. It would be interesting to know how to understand what makes an artform ‘socially acceptable’ and yet used for something that is opposite, and still does not make the artform non-acceptable. Drama for the project is a mainstream as well as a protest against mainstream. A detailing of this experience would help evolve the nuances of the public engagement model that the project aims to mainstream.

 Thirdly, one important miss among the list of stakeholders, I believe, is the institution of caregivers, who reside with the survivor, within home, within community or embedded in other institutions. The caregiver often is a survivor of as much of stigma as experienced by the mental health survivor, without actually the so-called luxury of even being identified as a victim or even potential victim. Often the caregiver is also in the spectrum of those perpetrating stigma. What it tells us is the need to find a community-driven or rather survivor-driven way of naming the categories for the project. The conventional terms, when used for categories, might leave out certain important stakeholders, just because they do not have ‘names’. Finally, the project, in its ethical consideration, has stated progressive ways of looking at ‘informed consent’, especially the space for re-consent. It also has special pathways to consent for mental health survivors and for others. In that sense, the process has been devised appropriately keeping the interest of specific identities in mind. It might be good to further problematize ‘informed consent’, in the way, ‘being informed’ often is influenced by the power relationships owing to the participation of faith leaders, traditional chief and also officials. All these are part of the baggage that comes with ’buy-in’ of stakeholders. In that sense, the consent is not necessarily informed, but influenced by the presence of so-called important people in the project. For example, when the project says that as part of an ethical process, “It was to be made clear that involvement in the focus group discussion or interviews is voluntary and should they choose not to take part, their relationship with the University of Zimbabwe or the Z factor project will not be affected.” This statement is important to ensure the non-consent is safe and does not do any harm. However, it is also important to understand that the participants may not necessarily be bothered about their future relationship with the project or university, rather their worry is about their relationship with current gatekeepers, who are participating in the project. These, I believe, are part of everyday ethics in the project settings, which one often ignores. The expectation from the article is not necessarily to provide the solution to the problem, but creating spaces for problematizing some of these businesses-as-usual.

 The kinds of Datasets not only show the rigour but also the complexities associated with the developing case for any public engagement models. The transparency of the project in disclosing these data in public domain shows the interest the project has in throwing itself open to constructive criticism. I am looking forward to evaluation findings and how the project was able to or struggled to reduce psychosis stigma; **and** to develop collaborative care between the traditional faith based and medical models. With respect to the public engagement model, I feel the current article itself provides a strong case for using art forms in a participatory way to, I would say, protest against the now-mainstream culture that stigmatizes psychosis.

 At the end, I want to make it clear that neither I am a doctorate in any discipline, nor have a record of having published in peer-reviewed journals. These comments are more from the domain of participatory action research practitioners, which as a member of Praxis Institute for Participatory Practices, I have been exposed to in a number of settings, including on community health.

Is the study design appropriate for the research question?

Yes

Is the rationale for, and objectives of, the study clearly described?

Yes

Are sufficient details of the methods provided to allow replication by others?

Partly

Are the datasets clearly presented in a useable and accessible format?

Yes

Reviewer Expertise:

Participatory research, Ethics, Community health

I confirm that I have read this submission and believe that I have an appropriate level of expertise to confirm that it is of an acceptable scientific standard.
